# Factors Influencing Meal Provision and Dietary Support Behaviour of Caregivers of People with Chronic Kidney Disease: A Cross-Sectional Study

**DOI:** 10.3390/nu16203479

**Published:** 2024-10-14

**Authors:** Georgina Lockwood, Lucimay Davey, Catherine McFarlane, Nicholas A. Gray, Hattie H. Wright

**Affiliations:** 1 Allied Health, Sunshine Coast Hospital and Health Service, Birtinya, QLD 4565, Australia; georgina.lockwood@health.qld.gov.au (G.L.);; 2 School of Health, University of the Sunshine Coast, Sippy Downs, QLD 4556, Australianicholas.gray@health.qld.gov.au (N.A.G.); 3 Renal Unit, Sunshine Coast Hospital and Health Service, Birtinya, QLD 4565, Australia; 4 Sunshine Coast Health Institute, Birtinya, QLD 4565, Australia

**Keywords:** diet therapy, family caregiver, food literacy, nutrition knowledge, meal provision, dietary adherence

## Abstract

Background/Objectives: Caregivers play an important role in supporting care recipients to navigate their health needs, including adherence to dietary recommendations, which are complex and multifaceted. This study aims to (i) describe the nutrition knowledge of caregivers of people with chronic kidney disease (CKD), and (ii) explore caregivers’ perceptions of their role in providing healthy meals and nutrition support for care recipients. Methods: A cross-sectional study design employed a multi-strategy research approach. Caregivers (n = 78) of people with stage 1–5 CKD or post-transplant were recruited from a single centre. Their nutrition knowledge was assessed quantitatively with the revised General Nutrition Knowledge questionnaire. Theory-informed semi-structured interviews of a sub-sample (n = 12) qualitatively explored caregiver perceptions. Results: Most caregivers were female (75.6%) and cared for a male care recipient (87%; aged 74 (66; 80) yrs.). The caregivers (75.6%) provided a meal ≥6 times/week to their care recipient and had moderate nutrition knowledge (66.1 (60.5; 73.9)%). Four themes emerged describing the caregivers’ perceptions of meal provision and nutrition support, including the following: (i) food literacy skills are valued; (ii) social support is important; (iii) caregivers’ sense of social responsibility; and (iv) the management of complex and multifaceted dietary needs. Conclusions: The caregivers had moderate nutrition knowledge; they wanted to provide healthy meals and support to their care recipients to adhere to dietary recommendations. Targeted, co-designed nutrition education programs for caregivers may enhance nutrition care delivery to people with CKD.

## 1. Introduction

Chronic kidney disease (CKD) affects more than 10% of the general population worldwide and has emerged as the leading cause of mortality globally [1,2]. CKD not only diminishes quality of life and life expectancy but is also exacerbated by the complex interplay of comorbidities that influence disease onset, management, and progression [3]. These comorbidities, such as hypertension, diabetes, and cardiovascular disease, not only increase the risk of developing CKD but also accelerate its progression [1,4]. Addressing comorbidities through lifestyle modifications, including a focus on diet, is a crucial component in the prevention and management of CKD [5,6]. Literature reports that low diet quality is frequently observed in people with CKD [7,8,9] and improved diet quality can achieve better disease control [10], prevent disease progression [11,12], and improved health outcomes [8,13]. In such cases the management of an individual’s health is achieved through promoting healthy dietary patterns and eating in line with general healthy eating principles and CKD-specific dietary recommendations. However, in many cases, dietary recommendations are poorly met or not sustained in the long-term [14]. 

The eating behavior and food choice of an individual is influence by a multitude of factors including biological predisposition, food experiences, personal factors (i.e., attitude, food literacy, and social norms), and environmental factors [15,16,17]. In a review by Lambert et al. [18] investigating dietary adherence in adults with end stage kidney disease, factors identified to be associated with dietary adherence include: socioeconomic, condition related, therapy related, health care team and system factors, as well as patient related factors. Social and family support was often identified by papers included in this review as an important patient related factor. Moreover, a case-control study in people receiving haemodialysis who had a family carer had higher adherence to dietary and fluid restrictions than those without a family carer [19]. 

Caregivers and family carers are often involved in the health care of people living with CKD [20,21]. Within this role caregivers often assume responsibility for an individual’s dietary needs [22]. Food provision that aligns with dietary recommendations for disease management is a task fulfilled by many caregivers and can pose significant challenges [23]. The renal diet has been reported to be overwhelming, frustrating and emotionally demanding by caregivers [24]. Foundational knowledge on healthy eating guidelines, dietary recommendations, and food choices may reduce caregiver burden in regards to providing dietary support [25]. The nutrition knowledge of caregivers of people with CKD is largely unknown and requires further investigation. Higher levels of nutrition knowledge have been associated with healthier food choices, particularly fruit and vegetable intake in adults [26]. A study involving Australian caregivers of individuals with intellectual disabilities revealed that these caregivers possessed lower nutrition knowledge when compared to the general public, potentially affecting their ability to plan and provide nutritious meals for their care recipients [27]. 

To better understand caregivers’ ability to provide dietary support, it is crucial to gain an understanding of their existing nutrition knowledge, as well as factors that contribute to their ability to provide appropriate support to their care recipients to adhere to dietary recommendations. This study therefore aimed to examine the nutrition knowledge of caregivers for individuals with CKD, as well as explore caregivers’ perceptions of their role to provide healthy meals and nutrition support to their care recipients. 

## 2. Materials and Methods

### 2.1. Study Design

We employed a pragmatic approach in this cross-sectional study using multi-stage research [28] to understand the multiple factors influencing caregivers’ provision of healthy meals and nutrition support to adhere to dietary recommendations. Quantitative data included sociodemographic information and level of nutrition knowledge collected with a survey. Semi-structured interviews explored caregivers’ thoughts, feelings and perspectives on healthy meal and nutrition support provision and allowed an opportunity for caregivers to provide novel perspectives [29]. Quantitative and qualitative data was combined to allow for a comprehensive understanding of the role of caregivers in supporting care recipients to adhere to dietary recommendations [28]. Caregivers of people with CKD attending renal outpatient clinics within the Sunshine Coast Hospital and Health Service (SCHHS) were recruited. Ethics was obtained from the Human Research and Ethics Committee of The Prince Charles Hospital (LNR/2018/QPCH/47886) and the Ethics Committee of The University of Sunshine Coast (A191219). 

### 2.2. Participants and Procedure

**Figure 1:**
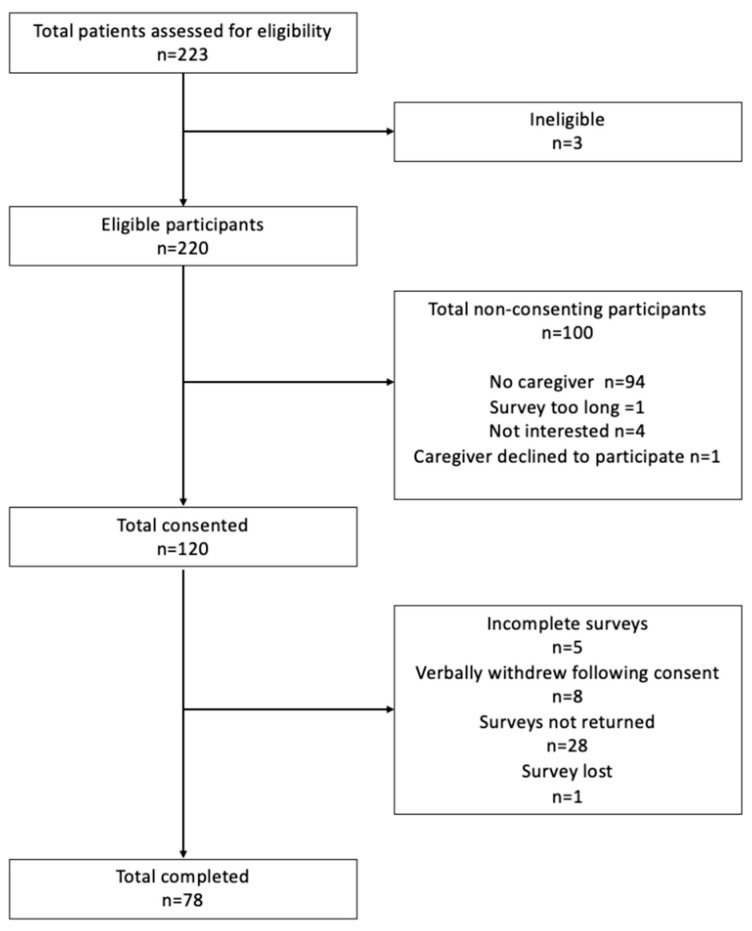
Recruitment procedure for final sample of included caregivers.

Individuals with CKD, not requiring dialysis, stages G3a to G5 and post-kidney transplant, were approached for recruitment via convenience sampling from two renal units in a regional health service between May and December 2021. Eligible patients were identified and provided with written information on the study to provide to their caregivers. The caregivers of those patients that provided written consent were approached to participate in the study. Written consent was obtained from the caregivers prior to their inclusion in the study. The exclusion criteria included patients receiving dialysis, <18 year of age, caregivers who were non-English-speaking, and those with cognitive impairment. The patients were screened for eligibility, and a final sample of 78 participants was included (see Figure 1). 

After their inclusion, the caregivers were asked to complete a survey regarding their sociodemographic characteristics and caregiving relationship, along with a nutrition knowledge questionnaire. The care recipient information was collected from hospital medical records, including age, gender, cause of CKD, transplant date, eGFR, comorbidity status, and smoking status.

### 2.3. Nutrition Knowledge Assessment

Nutrition knowledge was measured with the revised Australian general nutrition knowledge questionnaire (GNKQ). The GNKQ is a validated nutrition knowledge questionnaire for adults [30] which has been revised and validated to an Australian population based on the Australian dietary guidelines [31]. It is a self-administered, 117-item questionnaire comprised of four sub-sections assessing different areas of nutrition knowledge namely; (i) the Australian Dietary Guidelines (19 items scoring 19 points, (ii) food groups and food sources of nutrients (9 items, 53 points), (iii) knowledge of food healthy choices (10 items, 10 points), and (iv) the relationship between diet and disease (9 items, 35 points) with an overall maximum score of 117 [31]. In the current study, a percentage of less than 50% for the full questionnaire or any sub-section were deemed low or inadequate nutrition knowledge, a percentage of 50 to 74% as moderate nutrition knowledge, and a percentage of 75% or higher as a high level of nutrition knowledge. 

### 2.4. Caregiver Perceptions on Food Provision and Delivery of Nutrition Support

Semi-structured telephone interviews consisting of a series of open-ended questions, informed by the theory of planned behaviour [32], explored caregivers’ perceptions on their role in supporting their care recipients to follow dietary recommendations, and the provision of healthy meals to their care recipients. Questions were developed through a collaborative process involving dietitian clinicians with expertise in providing nutrition care to people living with CKD (GL and CM, over 40 years clinical experience as renal dietitians) and nutrition researcher with expertise in development of theory-informed interview questions (HHW), see Supplementary Materials Table S1 for interview questions. 

### 2.5. Analysis

#### 2.5.1. Quantitative Analysis

Statistical analysis was performed using IBM SPSS statistics package for Windows version 25 (Chicago, IL, USA). The data were analysed for normality, and appropriate descriptive statistics were used. The continuous data are presented as the means and standard deviations for the parametric variables and as the medians and interquartile ranges for the non-parametric variables. The categorical variables are presented as percentages of the total group and frequencies. 

Associations between the sociodemographic information (gender, age, and education level) and nutrition knowledge were explored using Spearman rank correlations. Relationships between the care recipients’ comorbidities and the caregivers’ nutrition knowledge on specific items were explored with chi-squared tests. A Wilcoxon signed ranks test explored the differences among the nutrition knowledge sub-sections. Student’s *t*-tests were used to explore differences in the nutrition knowledge scores between the caregivers that had received dietary advice from a dietitian and those that had not received dietary advice from a dietitian. Significance was set at *p* < 0.05.

#### 2.5.2. Qualitative Analysis

Individual telephone interviews were conducted at a predetermined mutually convenient time by one researcher (GL) in October 2022 and lasted between 9 and 24 min. The interviews were audio-recorded and transcribed verbatim. 

The transcripts were inductively analysed thematically according to the six-phase process of Braun and Clarke [33], namely familiarization with the data, initial coding, identifying themes, reviewing themes, defining and naming themes, and writing up the findings. 

For the data analysis, we employed a comprehensive and iterative approach. Following each interview, the interviewer (GL) reflected and recorded reflexive interview notes. Two researchers (CM and HHW) immersed themselves in the data by repeatedly listening to and transcribing the recordings. Three researchers (GL, CM, and HHW) independently coded two transcripts and compared and reconciled their codes to reach consensus. Using Microsoft Excel, coding continued with GL and CM independently analysing the remaining transcripts. The codes were organised into sub-themes, which were identified and defined based on their combined analysis. Finally, GL and CM (qualitative analysis experts) engaged in an iterative process to refine the sub-themes into final themes. Triangulation of the analysis through a review of the final themes by HHW (qualitative analysis expert) further strengthened the analysis. Additionally, reflexive interview notes informed the finalisation of the themes and sub-themes, with all researchers reaching agreement on the final themes. 

## 3. Results

### 3.1. Participant Characteristics

The caregiver characteristics are summarised in Table 1. The typical caregiver was female, aged 65 years or older, and cared for a spouse or partner. The majority always (64.1%) or usually (30.8%) bought groceries for their care recipient. Food preparation was completed by 75.6% of the caregivers ≥6 times a week for their care recipient and 3–5 times a week by 19.2%. The caregivers’ perceptions of their own health were varied, with 37.7% viewing it as very good or excellent, 36.4% as good, and 26% as fair or poor. 

The typical care recipient was a 74-year-old male presenting with multimorbidity (see Table 2). The most common self-reported cause of CKD was type 2 diabetes mellitus, followed by cardiovascular disease.

### 3.2. Nutrition Knowledge

Fewer than half of the caregivers (44.9%) reported to have received dietary advice on renal nutrition to support the management of their care recipient’s kidney disease. The top four sources of dietary advice were dietitians (32.1%), nephrologists (15.4%), general practitioners (14.1%), and nurse practitioners (10%). 

The caregivers’ overall nutrition knowledge was moderate, with significant (*p* < 0.05) differences in their nutrition knowledge on the sub-sections relating to ‘knowledge on food choices’ and ‘the relationship between diet and disease’ versus their knowledge on the ‘Australian dietary guidelines’ and ‘food groups and nutrients’ (see Table 3). 

No differences were found in the nutrition knowledge between caregivers who reported to have received dietary advice versus those who had not received dietary advice from a health professional (66.2% vs. 64.1%, *p* = 0.273).

#### Correlations

Positive associations were found between the female gender and overall nutrition knowledge (r = 0.317, *p* = 0.005), knowledge on the relationship between diet and disease (r = 0.382, *p* < 0.001), and knowledge on food groups and nutrients (r = 0.225 *p* = 0.048). Age was positively associated with knowledge on the relationship between diet and disease (r = 0.273, *p* = 0.016), and level of education was associated with knowledge on food groups and nutrients (r = 0.298, *p* = 0.01). 

The caregivers of people with type 2 diabetes as a comorbidity had adequate knowledge on carbohydrate-containing foods, whereas the caregivers of people without type 2 diabetes as a comorbidity did not (χ^2^ = 7.412, *p* = 0.025). Knowledge on the fibre and sugar content of food was, however, similar between the caregivers caring for care recipients with and without type 2 diabetes as a comorbidity. Similarly, no relationship was found for the adequacy of knowledge on the salt content of food products between the caregivers of care recipients with and without hypertension (χ^2^ = 0.724, *p* = 0.395). 

### 3.3. Caregiver Perceptions on Meal Provision and Providing Nutrition Support

Out of the n = 23 caregivers who expressed interest in participating in the qualitative component of the study, n = 12 were included (100% female, 67.5 (range of 38–74) yrs.). Three caregivers declined to partake in the semi-structured interviews when contacted by the research team, and eight did not respond after three email invitations. 

**Figure 2:**
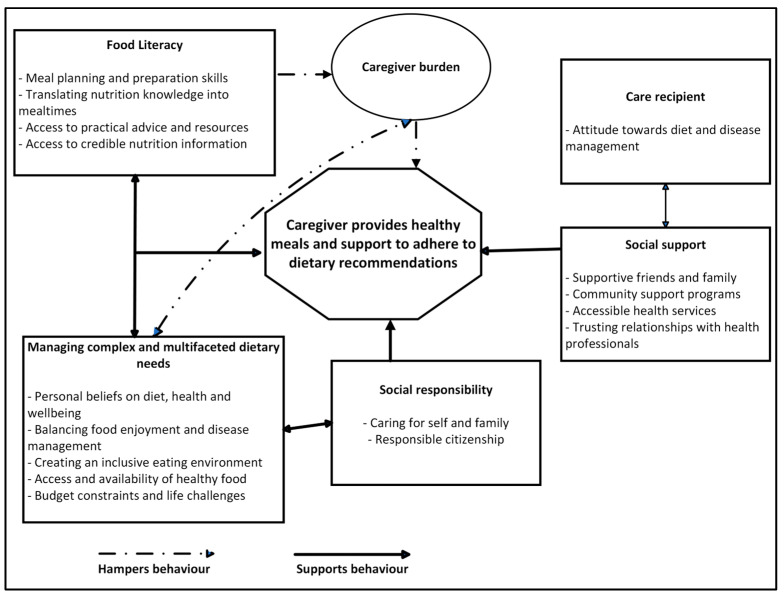
Factors influencing caregivers’ meal provision and dietary support behaviour.

Four themes emerged describing the caregivers’ perceptions on the provision of healthy meals and support to adhere to dietary recommendations, as follows: (i) food literacy skills are valued (meal planning and preparation, translating nutrition knowledge into mealtimes, access to credible nutrition information, and practical advice and resources); (ii) social support is important (timely and accessible health services, trusting relationships with health professionals, support from family, friends, and care recipient, and community support programs); (iii) the caregivers’ sense of social responsibility (caring for self and family, and responsible citizenship); and (iv) the management of complex and multifaceted dietary needs (access and availability of healthy foods, creating an inclusive eating environment, budget constraints and life challenges, balancing food enjoyment and disease management, personal beliefs about diet, and health and wellbeing). Figure 2 illustrates the interactions among key factors, which activate or hamper caregivers’ behaviour. Indicative quotes for each theme are illustrated in the Supplementary Materials, Table S2.

Food literacy skills were a key determinant of the caregivers’ ability to provide healthy meals. These include the ability to plan and prepare meals and translate nutrition knowledge into meal provision. In fact, those with strong food literacy were able to adapt recipes, utilise seasonal produce, and enable the provision of nutritious meals despite budgetary constraints.

“I could just make up something even if we haven’t gone shopping. I can put something on the plate still without worrying there’s nothing to eat so we always have something to eat.” Participant 10, 69 yrs.

and

“Sometimes it’s hard to make decisions when you don’t have a meal provided and you got to go to the shops and grab something on the run. I think that’s probably where things get a little bit difficult.” Participant 11, 38 yrs.

Nutrition knowledge and access to credible information provided a theoretical knowledge base in regard to healthy eating for CKD, as explained by participant 5, 72 yrs “… we have to look at that, fat and sugar content. Yeah, I always look at that sort of thing on the side of the food to make sure there’s not too much sugar and not too much fat. It’s hard.” Access to “written material”, practical resources such as “recipes” or “food lists”, and “cooking demonstrations” were all mentioned as important to inform meal provision. Advice and information provided from a renal specialist dietitian and other medical team members were highly valued.

“But these days there’s a lot of knowledge out there and for somebody being diagnosed, particularly younger people or an older person who isn’t much of a cook, to have somebody seriously say if he really wants that, then you do it this way…” Participant 4, 69 yrs.

Social support enabled caregivers to provide healthy meals. Caregivers found it easier to provide a healthy diet to their care recipient when the care recipient also valued this lifestyle choice and they had the support of family and friends. As explained by participant 9, 67 yrs, “We do it together really because we both enjoy those choices”. Consistent and positive reinforcement of dietary recommendations by all health professionals was reported to enhance care recipients’ motivation for adherence. However, inconsistent advice on the dietary management of CKD resulted in confusion and reduced confidence in meal provision.

“We had a sample meal plan done previously by a dietitian where it’s 80 g of protein a day, but doctor [name] doesn’t seem to value so much of the protein per day … he just seemed to brush it off, like not really that important. So, I don’t know. So, there’s a little bit of not sure what to do about that…” Participant 5, 72 yrs.

Timely access to health services was valued, and forming trusting relationships with health professionals enabled the care recipients to follow dietary recommendations and enhanced the caregivers’ confidence in supporting adherence to these recommendations. The need for greater access to reliable nutrition information was expressed, along with inclusion in nutrition education sessions and flexibility in the mode of service delivery, such as home visits, telehealth, and virtual education sessions to reduce travel time.

“I had access to support from the, either the nurses at the dialysis unit or the, the hospital. I could have easily just called somebody and talked to them if was feeling that I needed that support.” Participant 2, 68 yrs.

The caregivers exhibited a deep sense of social responsibility by consistently striving to provide nutritious meals for their care recipients and, where relevant, the rest of their family members. This social responsibility was likely underpinned by the fact that most caregivers were caring for a spouse, partner, and/or children.

“I encourage it [healthy meals]. Because if I’m doing it for myself and it’s okay to do it for my family, encouraging them all to eat healthy like portion controlling. It’s not just my care recipient who has to have portion-controlled food, it’s one of my others that I look after as well.” Participant 3, 51 yrs.

Responsible citizenship was demonstrated by supporting farmers’ markets to purchase “locally grown” food, and one care recipient expressed it was important to “look after” a new kidney post-transplant through supporting their care recipient to engage in health behaviours. 

Meal provision by the caregivers was recognized as a dynamic and intricate task due to the often complex and multifaceted dietary requirements of the care recipients. These requirements extended beyond kidney disease management to encompass the nutritional needs associated with other chronic conditions. The caregivers consistently emphasized the positive impact of following a healthy diet and adhering to dietary recommendations on overall health and well-being, as well as disease management.

“…weight control is a big thing because he seems to put tummy weight on very easily. He’s already got lupus and various other issues so adding another one like obesity on to it is probably not something we want to do. So just keeping him nice and healthy is the best option.” Participant 11, 38 yrs.

Additionally, the caregivers discussed juggling the dietary requirements and food preferences of other household members, budgetary constraints, and time factors. The use of “online meal delivery” services and healthy preprepared meals helped to provide healthy meals when the caregivers were time-poor. Budget and financial constraints influenced the types of food purchased by the care recipients, who emphasized the importance of strong food literacy, especially during times of financial hardship. In fact, budget constraints were viewed as a barrier to providing healthy meals.

“… it’s also budget control. Like, it depends on what we can afford as well. That often directs what type of healthy food we can have. Budget’s actually a big influence especially of late.” Participant 3, 51 yrs.

The caregivers were able to provide healthy diets for their care recipients by leveraging their awareness of community food programs and access to affordable food sources. These resources included homegrown produce from a “veggie garden”, affordable and “fresh produce” from local farmers markets, and community food assistance programs. Specific ideas and beliefs about what constitutes a healthy diet were expressed, and the types of food provided to care recipients were influenced by the caregivers’ personal food beliefs and preferences.

“…we always buy almost every time the same thing, lots of veggies and fruit and all that. My trolley is mainly just that and people just stare at us. I don’t know why. I feel embarrassed sometimes.” Participant 10, 69 yrs.

The importance of balancing food enjoyment with disease management was highlighted. An emotional burden of feelings of guilt when favourite foods were not allowed was expressed by the caregivers. This was managed by being mindful of incorporating favourite foods into the diet to enhance the quality of life and enjoyment of their care recipient.

“I mean we do indulge, but it’s planned, it’s organised and it’s small amounts.” Participant 4, 69 yrs.

## 4. Discussion

This study aimed to investigate the nutrition knowledge of the caregivers of people with CKD and explore their perceptions on healthy meal provision and the delivery of nutrition support to gain a better understanding of the factors influencing their ability to support their care recipients’ dietary needs. The key findings indicate that the caregivers’ overall nutrition knowledge was moderate, while their knowledge on healthy food choices was high. The female gender, older age, and a higher education level were all associated with greater nutrition knowledge. The key themes identified to influence caregivers’ meal provision and nutrition support behaviours highlighted food literacy, the ability to manage complex and multifaceted dietary needs, a sense of social responsibility, and social support as important determinants. 

Adequate nutrition knowledge is one of a multitude of factors underpinning healthy eating behavior [17]. The nutrition knowledge of this cohort of caregivers was moderate which is similar to other Australian community groups [27,31] and caregivers of people with dementia [34] but higher than carers of people with intellectual disability [27]. Knowledge on the Australian dietary guidelines (ADGs) as well as food groups and nutrients scored lower than other sub-sections on the nutrition knowledge survey in the current study. The ADGs are evidence-based population dietary guidelines promoting healthy eating patterns in order to reduce chronic disease risk [35] and manage non-communicable disease [36,37]. Adhering to healthy eating guidelines may reduce the risk of CKD and is promoted to manage early CKD to improve cardiometabolic health [8,38] and slow disease progression [39,40] amongst other health benefits [13,38]. Nutrition knowledge on food groups and nutrients provides insight into the ability of caregivers to identify key foods and nutrients for the management of not only CKD but also other chronic disease such as hypertension and diabetes [41,42]. Despite lower nutrition knowledge scores on the ADGs and food groups and nutrients sub-sections, caregivers scored high on their knowledge relating to diet-disease relationship. This indicates a sound understanding of the link between certain foods and risk of chronic disease including cardiovascular disease and obesity. Furthermore, their knowledge on this sub-section was higher compared to caregivers of people with dementia [34], likely due to the presence of comorbidities prior to development of CKD which also requires dietary management. In fact, sociodemographic factors such as specialist dietary needs, age, gender, and education was associated with nutrition knowledge which is in agreement with others [27,43]. Important to note was that male care recipients, younger age, and lower education level were associated with lower nutrition knowledge and highlights a need for targeted nutrition education and support programs for these caregivers. 

Evidence points to the importance of an adequate understanding of healthy eating patterns, food groups and sources of key nutrients by caregivers as it may influence the types of food provided as well as adherence of care recipients [44]. In fact, caregivers who valued the role of nutrition in the management of CKD and overall health in the current study had a positive attitude towards providing healthy meals to care recipients. Additionally, understanding the link between nutrition and disease management empowered caregivers to support and motivate their care recipients to make healthy food choices. Caregivers consistently highlighted the belief that adhering to dietary recommendations would have a positive impact on overall health and wellbeing and non-adherence results in negative health outcomes for their care recipients. Our findings are in accordance with others [45,46] where caregivers and/or care recipients agree adjusting dietary behavior are more likely when there is a strong belief in the health consequences associated with dietary adherence [14,47,48]. Therefore, knowledge on general healthy eating principles and dietary recommendations for the management of CKD is vital to empower and motivate caregivers to plan and provide appropriate meals and enhance adherence to dietary recommendations of care recipients. There is a scarcity of nutrition education programs targeted towards caregivers of adults living with chronic disease, such as CKD, despite it being a promising strategy to enhance both caregiver and care recipient dietary behavior, improve health outcomes, and reduce caregiver burden [25]. A hospital-based education and support program for caregivers caring for older adults showed promise to develop assertiveness, self-awareness and problem-solving skills of caregivers through the provision of a combination of information and education (including nutrition), sharing, and social support [49]. Further research into the nutrition education and support needs of caregivers of people with CKD is warranted as well as the development and evaluation of targeted nutrition education and support programs.

Food literacy and access to reliable nutrition information was another key determinant of caregivers’ perceived ability to adequately support care recipients to adhere to dietary recommendations for kidney health in the current study. Similarly, Rezaie [50] reported caregiver access to adequate nutrition information and dietetic services was vital to enable them to provide the required nutrition support for care recipients receiving hemodialysis treatment. The sentiment to involve caregivers in nutrition education sessions were echoed in people with early CKD. Carers were described as gatekeepers to change, playing a role in decision making on food choices, cooking and meal planning [45,51]. Cicolin et al. [19] found family carers improved dietary adherence in hemodialysis patients as carers provided patient support, filled knowledge gaps, and reminded them of dietary restrictions. In the current study, caregivers expressed the need for individualized and in-time access to credible nutrition information. The mode and format of delivery, as well as type of nutrition information required differed based on existing nutrition knowledge and skills. A systematic review evaluating educational interventions aimed at primary or secondary prevention of CKD found multifaceted educational interventions that are interactive and include individual as well as group participation was most promising to improve self-management of people living with CKD [52]. It can be argued that future interventions should include caregivers given their integral role in the translation of dietary recommendations at home. In fact, the need for caregiver inclusion in nutrition education session is not new [24] and seems to remain an unmet need. Donald et al. [23] identified key characteristics of desired self-management interventions through a qualitative exploration into the needs of people living with CKD and their caregivers. Timely access to credible, understandable, and meaningful information was highlighted as an unmet need and practical support from family carers was valued as key drivers of disease self-management. Of note is that caregivers in the current study spoke about the challenges faced to provide healthy meals in the face of rising living costs. Those reporting sound food literacy skills were able to overcome budgetary constraints as a hurdle to providing healthy meals. However, those reporting poor cooking skills and lacked confidence in translating nutrition information and recommendation to meal provision raised the need for practical nutrition resources and training sessions such as cooking demonstrations. Our findings highlight the need for nutrition programs co-designed by caregivers to enhance food literacy skills and ensure in-time access to individualized nutrition information, particularly in the current economic environment with rising food and living costs [14,53]. 

Most caregivers in our study were partners or spouses of their care recipients, positioning them not only as integral members of the care team but also as influential figures within the family dynamic, directly shaping social and subjective norms. Caregivers had significant involvement in grocery shopping and meal preparation highlighting their instrumental role in providing nutritional support to their care recipient. In fact, others have shown a positive attitude towards dietary change in people with CKD is associated with social expectations and social support [18,46]. Similar findings have been reported in other clinical populations. For example, an umbrella review on factors influencing diabetes self-management in adults, discussed the blanket success of social and cultural support in disease management in that positive social influences (roles and norms) were found to facilitate better diabetes self-management [47]. A study by Yehle et al. echoed the importance of social support to enhance dietary adherence when examining coronary heart disease patients’ views of dietary adherence and a supporting web-based tool [54]. A key theme that emerged in the care model was that social support makes dietary adherence easier for the patient [54]. Similarly, we found that social support in the form of caregivers can make dietary adherence easier for care recipients. In addition, caregivers play an important role in incorporate favorite foods while balancing dietary needs of care recipients, as well as considering the rest of the household’s dietary needs and preferences [14,45]. This approach ensures enjoyable mealtimes for all and contributes to a good quality of life, aligned with caregiver’s desire to balance dietary compliance with food enjoyment. Finally, consistent messages from health professionals regarding the role of nutrition in management of CKD was key to motivate care recipients to adhere to dietary recommendations, as well as enhance caregivers’ confidence in providing appropriate meals and nutrition support which is in agreement with the literature [23,24,46]. 

### Strengths and Limitations

The strengths of this study include combining quantitative and qualitative enquiries to gain a complete picture of the factors influencing caregivers’ abilities to provide healthy meals and dietary support, the use of a validated tool to assess the general nutrition knowledge and health literacy of caregivers, using theory-informed interview questions to describe the caregivers’ perceptions on meal provision and nutrition support, and following a robust qualitative analysis process. This study was undertaken in a single health service, which limits the generalizability of the findings. Due to the use of convenience sampling, selection bias may result, and the results should be interpreted with care. Most participants were white female caregivers over 60 years, which limits the generalizability to male or younger caregivers or carers from minority groups. Confirmation bias may occur during interpretation of the study data; however, research rigor was employed during the qualitative analysis using an iterative process and researcher triangulation in finalizing the qualitative themes. Two questions in the sub-section on knowledge on food choices of the general nutrition knowledge questionnaire were omitted by human error. One question was on the interpretation of the health star rating; however, there was another question on the same topic which captured the caregivers’ ability to interpret the health star rating. Another question was on label reading, asking the participants to identify the product with the highest energy content per 100 g. The relative percentage of the sub-section result and the overall nutrition knowledge result were adapted accordingly; however, the findings need to be interpreted with care, as knowledge on food choices may be positively skewed.

## 5. Conclusions

This study reveals that caregivers of individuals with CKD or renal transplant recipients exhibited moderate overall general nutrition knowledge and reported significant responsibility for healthy meal provision to their care recipient. Nutrition knowledge formed the theoretical basis from which meals were provided, while sound food literacy skills, alongside adequate social support, reduced the caregivers’ burden to provide healthy meals and provide the required dietary support. These findings present a valuable opportunity to leverage caregivers’ decision making, influence, and social support in helping care recipients to adhere to dietary recommendations. There is a need for targeted, co-designed nutrition intervention programs for the caregivers of individuals with kidney disease to empower them with the knowledge and food literacy skills to effectively assist their loved ones in managing their condition through dietary changes.

## Figures and Tables

**Table 1:** Sociodemographic characteristics of caregivers (n = 78).

Variable	Category	Findings ^1^
Age (years)	18–44	6.5 (5)
	45–64	27.3 (21)
	65+	66.3 (51)
Gender	Female	75.6 (59)
	Male	24.4 (19)
Main language spoken	English	98.7 (76)
	Other	1.3 (1)
Education Level	Primary school	4 (3)
	Secondary/high school	37.3 (28)
	Trade or certificate	21.3 (16)
	Diploma	24 (18)
	Degree	12 (9)
	Post-graduate degree	1.3 (1)
Income per week	AUD 0–750	42.9 (33)
	AUD 751–1430	29.9 (23)
	AUD 1431–2430	10.4 (8)
	Over AUD 2430	1.3 (1)
	Prefer not to say	15.6 (12)
Relationship to care recipient	Spouse/partner	87 (67)
	Daughter/son	1.3 (1)
	Parent	7.8 (6)
	Other	3.9 (3)
Marital status	Married/partnered	89.6 (69)
	Separated/divorced	6.5 (5)
	Single	3.9 (3)

^1^ Data are expressed as percentages of total group (frequency) for categorical data and as medians and interquartile ranges (25th; 75th) for non-parametric variables. AUD, Australian dollar.

**Table 2:** Care recipient health information and characteristics (n = 78).

Variable	Category	Findings ^1^
Age (yr)	-	74 (66; 80)
eGFR ^2^ (mL/min/1.73 m^2^)	-	27 (18; 42)
Gender	Male	75.6 (59)
	Female	24.4 (19)
CKD Cause	Diabetes	25.6 (20)
	Hypertension or vascular disease	24.4 (19)
	Glomerulonephritis	21.8 (17)
	Other	14.1 (11)
	Polycystic kidney disease	6.4 (5)
	Reflux neuropathy	3.8 (3)
	Obstruction	2.6 (2)
	Hereditary	1.3 (1)
Comorbidities	Hypertension	76.9 (60)
	Diabetes	42.3 (33)
	Ischaemic heart disease	29.5 (23)
	Cardiovascular disease	16.7 (13)
	Peripheral vascular disease	11.5 (9)
Multimorbidity ^3^	None	10.3 (8)
	One	32.1 (25)
	Two or more	57.7 (45)
Smoking status	Smoker	29.5 (23)
	Non-smoker	60.3 (47)
	Unknown	10.3 (8)
Kidney transplant recipient	Yes	15.4 (12)
	No	84.6 (66)

^1^ Data are expressed as percentages of the total group (frequency) for the categorical data and as medians and interquartile ranges (25th; 75th) for the non-parametric variables. ^2^ eGFR, estimated glomerular filtration rate. ^3^ Multimorbidity refers to the presence of two or more chronic conditions in a person at the same time.

**Table 3:** Caregiver overall nutrition knowledge and sub-section nutrition knowledge (n = 78).

Variable	Raw Score	Percentage
Overall nutrition knowledge ^1^	76 (69.5; 85.0)	66.1 (60.5; 73.9)
Sub-section 1: Australian dietary guidelines	12 (11; 13.25)	63.2 (57.9; 69.7) ^a^
Sub-section 2: Food groups and nutrients	33.5 (28; 39)	63.3 (52.8; 73.6) ^b^
Sub-section 3: Knowledge on food choices	7 (6; 8)	87.5 (75.0; 100.0) ^c^
Sub-section 4: Relationship between diet and disease	25 (23; 28.0)	71.4 (65.7; 80.0) ^d^

Data are expressed as medians and interquartile ranges (25th; 75th). ^1^ Total score out of 115 due to omission of two questions in sub-section 3, sub-section 1 scored out of 19 points, sub-section 2 scored out of 53 points, sub-section 3 scored out of 8, and sub-section 4 scored out of 35 points. ^a and b^ *Z* = −2.000, *p* = 0.046; ^a and c^ *Z* = −6.480, *p* = 0.000; ^a and d^ *Z* =−3.205, *p* = 0.001; ^b and c^ *Z* = −6.640, *p* = 0.000; ^b and d^ *Z* = −5.192, *p* = 0.000; ^c and d^ *Z* = −5.965, *p* = 0.000.

## Data Availability

The data supporting the reported results are available upon reasonable request and in accordance with the ethical principles.

## Supplementary Materials

The following supporting information can be downloaded at https://www.mdpi.com/article/10.3390/nu16203479/s1, Supplementary Materials Table S1: Semi-structured interview questions; Table S2: Identified themes describing caregiver perceptions on meal provision and nutrition support to care recipients with indicative quotes.
