# Supplementary material for: Factors Influencing Meal Provision and Dietary Support Behaviour of Caregivers of People with Chronic Kidney Disease: A Cross-Sectional Study
Source: Nutrients. 2024 Oct 14;16(20):3479. doi: 10.3390/nu16203479 (PMC11510062; doi:10.3390/nu16203479)
Supplement: Supplementary file 1 [file nutrients-16-03479-s001.zip › nutrients-3213914-supplementary.pdf]

Supplementary materials

Table S1. Semi-structured interview questions

Questions on Attitude

- How do you see your role in providing healthy meals/snacks to you care recipient?
- What do you see as the **advantages or benefits in** providing healthy meals and or snack to your care recipient?
- What do you see as the **disadvantages** of providing healthy meals/snacks to your care recipient?

Questions on Subjective norm

- Who **approves** of or think you should provide healthy meals/food to your care recipient?
- Who **disapprove** or think you should not provide healthy meals/food to your care recipient?

Questions on Perceived behavioural control

- How confident do you feel to provide healthy meals/snacks to your care recipient?
- Can you describe the factors that influence the foods you provide to your care recipient?
    - Probe: Are there any specific considerations you take in relation to CKD when providing food to your care recipient?
- What makes it **easy** for you to provide healthy meals/snacks to your care recipient?
  - Probe: What support would help you to provide healthy meals/snacks to your care recipient?
- What makes it **hard** for you to provide healthy meals/snacks to your care recipient?
  - Probe: How does your own knowledge on nutrition influence the food that you provide to your care recipient?

Table S2. Identified themes describing caregiver perception on meal provision and nutrition support to care recipients with indicative quotes (n=12).

Main themes	Sub-themes	Indicative quote
**Food literacy skills are valued**	Meal planning and preparation	• "Preparation, sourcing the food, and making sure that the ingredients are here at home if they wish to get up and do it for themselves." Participant 3, 51 yrs.<br>• "We make sure that we try and prep those things because we're a busy little household." Participant 11, 38 yrs.<br>• "I enjoy cooking, so it's never a problem." – Participant 8, 69 yrs.<br>• "I could just make up something even if we haven't gone shopping.." Participant 10, 69 yrs.
	Translating nutrition knowledge into mealtimes	• "Being able to go to shops and make informed choices about how much meat that we have so we are not overindulging…" Participant 3, 51 yrs.<br>• "…certainly in the choices but also in the way they're cooked, making the meals a little bit more bland. Perhaps having some frozen vegetables of course because they're sometimes more nutritious. How I might cook potatoes for example might be different to how I would normally do them." Participant 9, 67 yrs.<br>• "Sometimes it's hard to make decisions when you don't have a meal provided and you got to go to the shops and grab something on the run. I think that's probably where things get a little bit difficult." Participant 11, 38 yrs.<br>• "So, we have to look at that, fat and sugar content. Yeah, I always look at that sort of thing on the side of the food to make sure there's not too much sugar and not too much fat. I try to, it's very hard." Participant 5, 72 yrs.
	Access to credible nutrition information	• "[Care recipient's] first urologist said, "No, go and see a dietitian and she'll tell you what to eat." But they weren't educated, I'm sorry, I

		mean they weren't specialists, they were just a dietitian." Participant 4, 69 yrs.<br>• "No, nobody's ever really told me precise food, sort, that I should or shouldn't use." Participant 6, 74 yrs.<br>• "…in the hospital after he had the transplant, it was definitely the dietitian that helped us out there and um yeah, she was very good." Participant 2, 68yrs.<br>• "The doctor, the hospital. Sometimes after he was hospitalized they give us a list of the things that, I mean we have to follow. The diet and stuff." Participant 12, 61 yrs.
	Practical advice and resources	• "But these days there's a lot of knowledge out there and for somebody being diagnosed, particularly younger people or an older person who isn't much of a cook, to have somebody seriously say if he really wants that then you do it this way..." Participant 4, 69 yrs.<br>• "…a refresher book, books, written material, some good advice of course from doctors it would be good to remember on what to do. It is not easy with his condition." Participant 12, 61 yrs.
**Social support is important**	Timely and accessible health services	• "It's good if somebody got this written material to keep refreshing, to keep refresh my memory. And also, if somebody can give me the reference people that I can contact if I need to." Participant 12, 61 yrs.<br>• "So, because he's indigenous … he's part of the chronic disease team …they often get encouragement and support from the indigenous side of it." Participant 3, 51 yrs.<br>• "I had access to support from the, either the nurses at the dialysis unit or the, the hospital. I could have easily just called somebody and talked to them if was feeling that I needed that support." Participant 2, 68 yrs.
	Trusting relationships with health professionals	• "We had a sample meal plan done previously by a dietitian where it's 80 grams of protein a day, but doctor [name] doesn't seem to value so much of the protein per day … he just seemed to brush it off, like not really that important. So, I don't know. So, there's a little bit of not sure what to do about that…" Participant 5, 72 yrs.

		• "But he [nephrologist] says, "Doesn't matter what you do with your diet it's not going to make any difference." Participant 4, 69yrs.<br>• "…in the hospital after he had the transplant, it was definitely the dietitian that helped us out there and um yeah, she was very good." Participant 2, 68 yrs.
	Care recipient's support	• "We do it together really because we both enjoy those choices." Participant 9, 67 yrs.<br>• "My son often cracks up about it, but not the care recipient, he'll just eat what's coming." Participant 3, 51 yrs.<br>• "…because we would tend to eat the same things, he doesn't feel like, I've got to do something special for him." Participant 2, 68 yrs.
	Supporting family and friends	• "I've only got one daughter …if we eat over there, she provides for him as well." Participant 7, 62 yrs.<br>• "…my friends said, "My God, I don't know how you fellows can eat like that. It's too healthy for us." Participant 10, 69 yrs.<br>• "most of um our family and friends realize this now and so they will, if we're visiting or whatever, they realize this and they know that he can't have certain things, and that, they're accommodating as well." Participant 2, 68 yrs.
	Community services and programs	• "I've signed up to places like Carers Gateway and the place where I used to volunteer at Suncoast Care, and having access to places like that, that help low-income families." Participant 3, 51 yrs.<br>• "Online delivery. Like so I could do my shopping during the day at work, um, and just have it delivered at night when I got home." Participant 1, 54 yrs.
**Caregivers' sense of social responsibility**	Caring for self and family	• "I don't mind [making dinner for 6 people]. You know what I mean? Taking care of my family." Participant 3, 51 yrs.<br>• "…when you do it at home [meal preparation] you know what it is, you know it's in a clean kitchen and with kidneys you're very prone, you get one little bug and that's it, you're sick as anything." Participant 7, 62 yrs.<br>• "I'm better at doing things for other people than I am at doing things for myself." Participant 1, 54 yrs.

		• "I think it [healthy meals] benefits both of us really because, you know we're eating healthy food. And I actually only have one kidney as well, so you know, I want to look after mine." Participant 2, 68 yrs.
	Responsible citizenship	• "We've been down this track of waiting for a long time for a kidney and now we've got one we are going to look after it." Participant 2, 68 yrs.<br>• "There's markets on the weekend and a lot of that food also is relatively locally grown …." Participant 4, 69 yrs.<br>• "There's a fairly good community here and a lot of them are growers. So, we shop around from one another …" Participant 8, 69 yrs.
**Meal provision is complex and dynamic**	Access and availability of healthy foods	• "Noticing that when they're in hospital they often don't get an adequate diet for them…" Participant 3<br>• "Go in the garden, get all the veggies and you have a nice meal." Participant 10, 69 yrs.<br>• "Maybe sometimes the longevity of some of the food that we buy. [makes it hard to provide healthy meals] Maybe if it's not used quickly it expires or if it's not stored correctly then obviously it goes a bit funky." Participant 11, 38 yrs.
	Budget constraints and life challenges	• "Sometimes it comes down to life circumstances to make it hard. But I tried to do the best I could by creating healthy meals. Like, one pot wonders and stuff like that." Participant 3, 51 yrs.<br>• "I don't like paying some of the prices where the prices went recently…" Participant 4, 69 yrs.<br>• "…mine [cabbage] is thriving at the moment. Because it's expensive, they went right up, the price of them. Terrible." Participant 7, 62 yrs.
	Creating an inclusive eating environment	• "…trying to eat healthy as a family, rather than just specifically targeting them." Participant 3, 51 yrs.<br>• "Because I love healthy, I love vegetables. I'd hate to have him as a child. It must have been a bugger for his mum. Fruits and things like that. But I love fruit and vegies and things like that." Participant 7, 62 yrs.

		• "…it is only the care recipient as sometimes he doesn't get what he wants to eat. I just sometime, you know give him what he wants even if it is not healthy." Participant 12, 61 yrs.
	Managing multifaceted dietary needs	• "Then if we go somewhere, that's when he loves to be naughty. He suffers for it… in a couple of days.." Participant 7, 62 yrs.<br>• "…that's not to say that we don't lash out and enjoy a pizza or beautiful pasta...we have a very good balance, everything in moderation." – Participant 9, 67yrs.<br>• "I guess we try and prevent things that he can't have. We don't eat much fatty foods or something that would do harm to his kidney." Participant 10, 69 yrs.<br>• "I just try to make everything. Even if he likes meat for instance, I just try to cut off a little bit. Yeah, in moderation. I mean like everything in moderation you know." Participant 12, 61 yrs.
	Personal beliefs about diet, health, and wellbeing	• "Yeah, that's what it [eGFR] – it's hovering around 24, so we don't want it to go any lower. So, that's what we look at and that's why I'm pushing not too much sweets and plenty of water." Participant 5, 72 yrs.<br>• "The healthier the food that goes in, the healthier the body is." Participant 4, 69yrs.<br>• "..to keep his um, nutrition levels up, so he's getting good nutrition and um, yeah just to keep him healthy."- Participant 2, 68 yrs.<br>• "I just make sure it's [diet] healthy. Just common sense. It just helps [care recipient name] with his kidney disease." Participant 6, 74 yrs.
